# Challenges and opportunities targeting mechanisms of epithelial injury and recovery in acute intestinal graft-versus-host disease

**DOI:** 10.1038/s41385-022-00527-6

**Published:** 2022-06-02

**Authors:** Suze A. Jansen, Edward E. S. Nieuwenhuis, Alan M. Hanash, Caroline A. Lindemans

**Affiliations:** 1 Division of Pediatrics, University Medical Center Utrecht, Utrecht, The Netherlands; 2 Department of Stem Cell Transplantation, Princess Máxima Center for Pediatric Oncology, Utrecht, The Netherlands; 3 University College Roosevelt, Utrecht University, Middelburg, The Netherlands; 4 Departments of Medicine and Human Oncology & Pathogenesis Program, Memorial Sloan Kettering Cancer Center and Weill Cornell Medical College, New York, NY USA

## Abstract

Despite advances in immunosuppressive prophylaxis and overall supportive care, gastrointestinal (GI) graft-versus-host disease (GVHD) remains a major, lethal side effect after allogeneic hematopoietic stem cell transplantation (allo-HSCT). It has become increasingly clear that the intestinal epithelium, in addition to being a target of transplant-related toxicity and GVHD, plays an important role in the onset of GVHD. Over the last two decades, increased understanding of the epithelial constituents and their microenvironment has led to the development of novel prophylactic and therapeutic interventions, with the potential to protect the intestinal epithelium from GVHD-associated damage and promote its recovery following insult. In this review, we will discuss intestinal epithelial injury and the role of the intestinal epithelium in GVHD pathogenesis. In addition, we will highlight possible approaches to protect the GI tract from damage posttransplant and to stimulate epithelial regeneration, in order to promote intestinal recovery. Combined treatment modalities integrating immunomodulation, epithelial protection, and induction of regeneration may hold the key to unlocking mucosal recovery and optimizing therapy for acute intestinal GVHD.

## Introduction

**Fig. 1:**
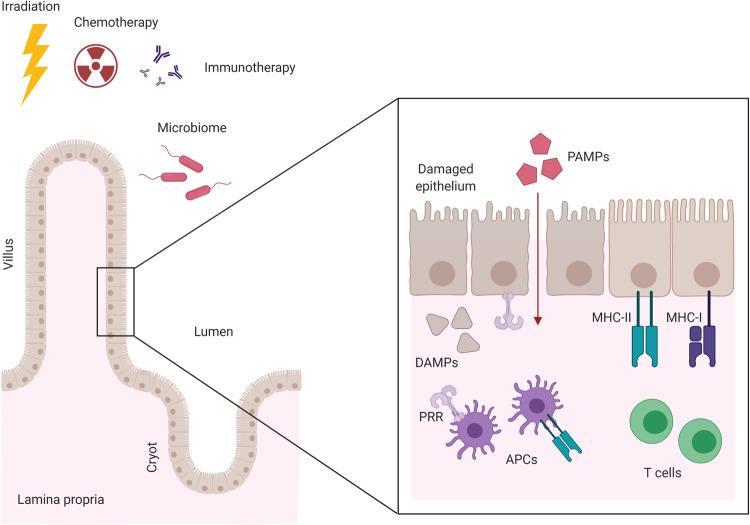
The pivotal role of the intestinal epithelium and epithelial damage in GVHD onset. Irradiation, chemotherapy, and/or immunotherapy used in the conditioning regimen before HSCT damages the intestinal epithelial cells and disrupts barrier protecting the recipient from luminal pathogens. Translocating pathogen-associated molecular patterns (PAMPs) and released damage-associated molecular patterns (DAMPs) bind to their corresponding pattern recognition receptors (PRRs) and activate the innate immune system, including professional antigen presenting cells (APCs). Antigen presentation by APCs, including the intestinal epithelium, lead to the propagation and activation of alloreactive T cells, which cause further damage through cytokine- and cell–cell-mediated toxicity in the then developed GVHD. Created with BioRender.com.

**Fig. 2:**
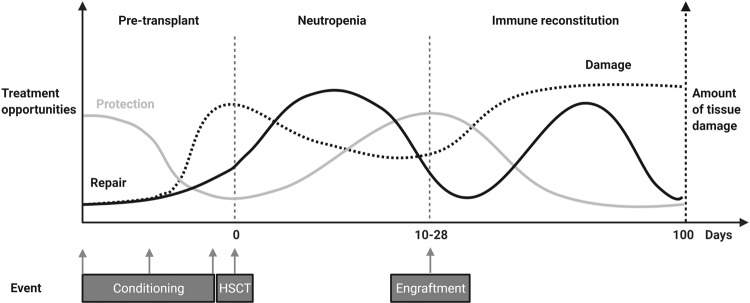
Opportunities for intestinal protection and repair over the course of HSCT. Damage to the intestinal epithelium over the course of HSCT occurs in different phases. As such, opportunities for protection against the insult and repair of the injury occur in parallel, rather than after the fact. The timing of these different approaches will be crucial, since certain treatment opportunities may have pleiotropic effects on other cell types at different time points during the posttransplant period. Created with BioRender.com.

Damage to the gastrointestinal (GI) tract is a common occurrence following allogeneic hematopoietic stem cell transplantation (allo-HSCT)^1,2^. Several factors are thought to contribute to this damage, including pretransplant conditioning, posttransplant activation of alloreactive T cells, and both tissue-targeted and immunomodulatory effects of the intestinal microbiota (Figs. 1, 2). Before transplantation of a donor allograft, the recipient receives chemotherapy ± irradiation conditioning to kill residual malignant cells, weaken the recipient’s immune system, and create space for donor hematopoietic engraftment. However, the required pretransplant conditioning can also cause significant damage to cycling cells in the epithelial gut lining, resulting in mucositis and disruption of the mucosal barrier. Impaired barrier function leads to exposure of the basolateral intestinal epithelial cell (IEC) membranes and lamina propria leukocytes to luminal contents. Activation of the immune system in this context may cause the development of acute Graft-versus-Host Disease (aGVHD). In aGVHD, transplanted donor T cells recognize antigens in the recipient and launch an inflammatory attack against the recipients’ tissues such as the skin, intestines, and liver^1,3,4^. Despite prophylactic immunosuppression and careful HLA-matching, ~30–50% of allo-HSCT patients develop GVHD symptoms, half of which include significant GI tract involvement manifesting in nausea, anorexia, and diarrhea. In addition, poor barrier function contributes to potentially life-threatening bloodstream infections in these immunocompromised patients. The immunosuppression and high dose corticosteroids necessary for treatment of GI-GVHD provide additional potential complications, even when GVHD can be successfully treated. As such, GI-GVHD remains an important cause of transplant-related morbidity and mortality^1,2^.

Current GI-GVHD treatment focuses mainly on suppressing posttransplant aberrant immune responses with corticosteroids^3,5^, but this approach is often ineffective. Up to 50% of patients develop steroid-refractory (SR)-GVHD and require additional treatment^6^. The Jak1/2-inhibitor Ruxolitinib (Rux) is currently the only FDA-approved treatment for SR-GVHD^7^. Other second and third-line therapies lack consistent demonstrated benefit, and are mostly based on providing additional immunosuppression. This causes most GVHD therapeutic approaches to be accompanied by an increased risk of infection and a potentially reduced graft-versus-leukemia (GVL) effect.

An evolving understanding of intestinal homeostasis and its related epithelial constituents has led to new treatment opportunities that aim to protect the intestines peri-transplant, without impairing the recovery of physiologic immune function posttransplant. It has also become increasingly clear that the intestinal epithelium is not only a direct target of GVHD-associated damage, but in addition may take part in the development and propagation of the disease, and possibly in its resolution as well^8^. In this review we focus specifically on the role of the intestinal epithelium and epithelial injury in GVHD initiation, and how it can be protected from transplant-associated insult. Furthermore, strategies to promote epithelial restoration by improving regeneration and augmenting posttransplant epithelial recovery are discussed, both through regulating epithelial-intrinsic constituents as well as factors supplied by the microenvironment.

### Preventing intestinal epithelial damage

#### Minimizing conditioning-induced injury

The intestinal epithelial barrier forms the first line of defense between the lumen and the underlying immune system in the gut^9^. As such it protects the recipient from harmful gut contents, including pathogens. The barrier is formed by the plasma membranes of a single layer of IECs that are tightly connected with tight junctions (TJ). The IEC lining is covered with a protective, extracellular layer of mucus produced by epithelial Goblet cells, which inhibits direct contact between the IEC and gut luminal particles and bacteria. The mucosal immune cells are present within the epithelial compartment, as well as in the lamina propria below, and in designated lymphoid regions called Peyer’s patches. In addition to providing a physical barrier, the epithelial cell layer is essential for the absorption and transport of nutrients and water. Both integrity and functionality of the epithelial barrier are crucial elements in the course of transplant, as loss is associated with systemic infections, severe GI symptoms like anorexia/diarrhea and poor outcome in general^2^.

Pretransplant conditioning is recognized as an early insult to this barrier integrity^10^. In both preclinical models and human studies overall aGVHD severity is associated with the intensity of the pretransplant conditioning regimen^11,12^. In mice, high intensity regimens were associated with reduced mucus layer thickness and the presence of bacterial RNA in the colon lamina propria^13^. Indeed, total body irradiation (TBI) and chemotherapy treatment led to significant leakage of orally administered FITC-labeled dextran into the bloodstream, indicating consequential compromise of the epithelial barrier^14^. In a study by Nalle et al. preconditioning-induced damage to the epithelium was even required to induce aGVHD in a MHC-matched minor-histocompatibility-antigen (miHA)-mismatched transplantation^10,15^. In humans, compromised epithelial integrity, as measured by ^51^Cr-EDTA absorption, has been documented in association with myeloablative regimens even 14 days after stem cell infusion^16,17^. Conditioning toxicity is also associated with the release of pro-inflammatory cytokines in the GI tract, which can contribute to GVHD development^11,18,19^. In particular, TBI appears to be associated with both a higher aGVHD incidence^20^ and treatment-related mortality^21^.

The implementation of reduced intensity conditioning (RIC) regimens has decreased conditioning-associated tissue toxicity and enabled older and more fragile patients to undergo allo-HSCT. Regimens typically include an alkylating agent like busulfan (Bu), melphalan or cyclophosphamide (Cy) and a purine analog such as fludarabine (Flu), with or without low-dose TBI. Clinical GI toxicity of RIC transplants is reported to be moderate^22,23^, and the associated intestinal epithelial damage^16,17^ and mucositis^24^ have been found to be less severe. The occurrence of aGVHD after RIC is reduced as well^25^. Additionally, more favorable combinations of agents have been applied to this approach. For example, in reducing the number of combined alkylators, Bu/Flu has a more favorable toxicity profile than Bu/Cy in patients across HSCT indications, while still providing a myeloablative regimen^26–28^.

More recent developments in conditioning regimen tolerability have focused on tailored dosing based on chemotherapy plasma levels of the individual patient to reduce exposure and consequent epithelial damage. This concept is known as reduced toxicity conditioning (RTC). The superiority of pharmacokinetic (PK)-directed dosing for intravenous Bu in adults has been established for over a decade with both enhanced safety^29^ and efficacy^30^. Similar results were found in children^31,32^. Additionally, Flu exposure, as calculated by a PK-model, was recently retrospectively demonstrated to be a strong predictor of HSCT survival in adults^33^. In conclusion, PK-directed dosing of favorable chemotherapeutic combinations will hopefully further reduce conditioning-related toxicity and development of acute GVHD in the near future.

#### Preventing deleterious responses to PAMPS and DAMPS

Upon intestinal barrier breach, translocating pathogen-associated molecular patterns (PAMPs) and tissue-released damage-associated molecular patterns (DAMPs) are recognized by pattern recognition receptors (PRRs), which activate the innate immune system. Concurrently, the development of alloreactive responses can be initiated^34–36^. As such, many DAMPs, PAMPs and corresponding PRRs have been implicated in the development of GVHD, and multiple approaches have been taken to dampen the response at this level (Table 1). Firstly, scavenging or breaking down the PAMPs and DAMPs would ascertain they do not reach the target immune cell. Examples are treatment with anti-LPS^37^, HSP90-inhibitor 17AAG^38^, locked nucleic acid anti-miRNA-29a^39^, uricase for uric acid^40^, apyrase for ATP^41^, NecroX-7 for HMGB1 blockade^42^ and alpha-1-antitrypsin (AAT) targeting heparin sulfate^43^, all of which have been shown to reduce GVHD in mouse models and AAT in addition SR-GVHD in humans^44^. Secondly, binding of DAMPs to target immune cells can be blocked, e.g., by P2X7R antagonists, blocking ATP binding^41,45^, or anti-TIM-1 monoclonal antibodies, inhibiting binding to phosphatidylserine on apoptotic cell debris^46^. Thirdly, responsiveness of immune cells to DAMPS could be modulated. MicroRNA-155 deficiency in host DCs protected against GVHD through reduced purinergic receptor and inflammasome-associated gene expression, and concurrent reduced IL-1β release^47^. Inhibiting microRNA-155 with antagomir could be a promising new approach. Contrarily, Siglecs play a crucial role in mitigating specifically DAMP-induced immune responses^48,49^. Following conditioning-mediated tissue damage, the interaction of Siglec-G on host APCs with the glycoprotein CD24 on T cells was essential for GVHD protection in both a MHC-matched and mismatched mouse model^50^. Enhancing the Siglec-CD24 interaction with a CD24-Fc fusion protein mitigated GVHD in experimental GVHD^50,51^. The results of a phase II trial testing the safety of CD24-Fc for the prevention of aGVHD following myeloablative allo-HSCT are expected shortly.

**Table 1:** All DAMPS/PAMPS implicated in GI-GVHD and targeted therapy options.

Receptor	DAMP/PAMP	Signaling pathway	Effect on GVHD	Therapeutic options	Ref.
TLR3	dsRNA	TRIF	=	–	^215^
TLR2/4	HMGB1	MyD88	−	NecroX-7	^42^
TLR4	LPS	MyD88/TRIF	−	Anti-LPS	^37^
TLR4	Heparan sulphate	MyD88	−	AAT	^43,44^
TLR4	S100 proteins	MyD88	−	–	^216^
TLR4/CD14	HSP90	MyD88	−	17AAG	^38^
TLR5	Flagellin	MyD88	+	Flagellin treatment	^217^
TLR7/8	ssRNA MiR29a	MyD88	−	locked nucleic acid anti-miRNA-29a	^39,218^
TLR9	Bacterial DNA	MyD88	−	–	^54,184^
cGAS	Bacterial DNA	STING	+	DNA treatment	^184^
RIG-I	dsRNA	MAVS	+	3pRNA treatment	^184^
Caspase-11	LPS	Pyroptosis/NLRP3	−	–	^56^
?	Uric acid	NLRP3	−	Uricase	^40^
P2X7	ATP	NLRP3	−	Apyrase P2X7R antagonists	^41,45^
NOD2	Eg MDP	NLRC	+	–	^190^
?	?	NLRP6	−	–	^52^
TIM	Phosphatidylserine	?	−	Anti-TIM	^46^
ST2	IL-33	MyD88	−	ST2-Fc treatment	^219^

*+* Alleviating, *=* No effect, *−* Worsening.

Additionally, targeting innate signaling pathways downstream of PRRs might be a future therapeutic approach^52–56^. Interestingly, host TLR deficiency was found to be protective against GVHD in murine studies^53,54^, but inhibition of TLR and inflammasome pathway signaling (via MyD88 and TRIF) only in host hematopoietic cells did not reduce GVHD^55^. This suggests a GVHD-promoting role of non-hematopoietic tissue signaling. More importantly, deficiency of TLR9^54^ and NLRP6 inflammasome^52^ was protective only when it was restricted to the non-hematopoietic compartment, indicating that TLR signaling at the tissue level, which includes the intestinal epithelial compartment, may be a future target for GVHD reduction. Despite the numerous possibilities of molecules and pathways to block, only very few damage-modulating agents have led to successful clinical trial results. Probably, the concurrent involvement of many different damage molecules as well as redundant downstream signaling pathways, make achieving significant clinical improvements by targeting just one molecule unlikely. Alternatively, involved molecules and signaling pathways may have concurrent roles in the resolution of GVHD, and targeting them would abrogate this, giving no net improvement.

#### Preventing cell death within the epithelial compartment

**Fig. 3:**
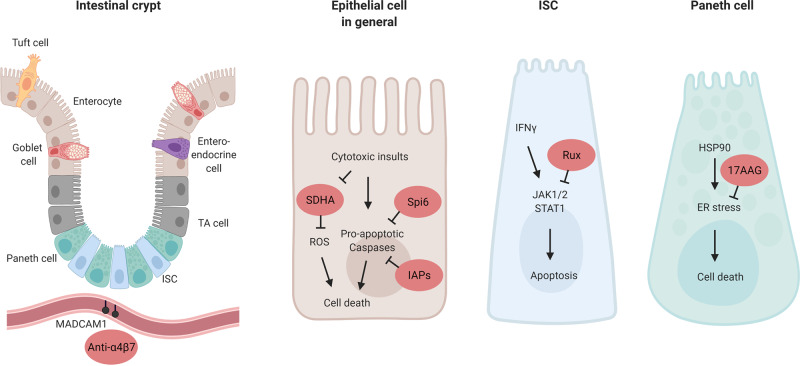
The intestinal crypt as a GVHD target, and mechanisms of protection. The intestinal epithelium is maintained by intestinal stem cells (ISCs) which reside at the base of intestinal crypts, interspersed between their supportive Paneth cells (PCs) in the small intestine. Along the crypt-villus axis the ISCs differentiate into transit amplifying (TA) cells and their destined lineage, including absorptive (e.g., enterocyte), secretory (e.g., PC, Goblet cell, Tuft cells) and enteroendocrine cells. In the vasculature near the intestinal crypt the addressin MAdCAM-1 is expressed, which binds α4β7-integrin expressed on gut-directed immune cells. Several approaches of protection at the level of the intestinal epithelial cell in general, or in addition at the ISC and PC level specifically, are indicated in red. A4β7 blockade inhibits the influx of T cells into the lower crypt regions of the small intestine; the serine protease inhibitor Spi6 present in the epithelium protects against GVHD-induced damage, possibly through inhibition of caspase 3/7; intestinal epithelial Inhibitor of Apoptosis Proteins (IAPS) inhibits the function of pro-apoptotic caspases; the SDHA enzyme is reduced in IECs after allo-T cell insult, increasing reactive oxygen species (ROS) levels; Ruxolitinib (Rux) inhibits JAK1/2-STAT1 signaling, relieving interferon (IFN)-y induced epithelial apoptosis; and 17AAG was reported to suppress ER stress and thereby cell death in a.o. Paneth cells. Created with BioRender.com.

Under homeostatic conditions, the intestinal epithelium is continuously regenerated by stem and progenitor cells that are present within the crypt region (Fig. 3). While a point of longstanding debate, work from the last 15 years has identified Lgr5-expressing crypt base columnar (CBC) cells as intestinal stem cells (ISCs) capable of giving rise to all other cell types of mouse and human intestinal epithelium in vivo and ex vivo^57–59^. Olfm4 is another marker identifying ISCs^60^ in humans and in mouse small intestine. ISCs are maintained by both secreted and membrane-bound molecules of surrounding cells, together constituting the ISC niche^61^. These niche cells include Paneth cells (PCs), which lie interspersed between CBC ISCs at the bottom of the crypt and promote stemness through the release of Wnt3 and EGF, which bind to their respective receptors Frizzled–LRP5–LRP6 complex and ERBB1 on CBCs^62,63^. PCs also express Notch ligands DLL1 and DLL4 on the cell-surface that directly interact with ISC Notch receptors such as NOTCH1 to maintain stemness and inhibit differentiation into secretory-cell lineages^62^. Crypt-adjacent stromal cells also promote ISC maintenance through the secretion of Wnts^64^ and R-spondins (Rspo)^65,66^. Rspo-binding of LGR5 on the ISC potentiates Wnt signaling by phosphorylation and stabilization of β-catenin in the cytoplasm, thus promoting subsequent translocation to the nucleus^67^. To protect ISCs from mesenchymal-derived epithelium-maturating BMP2 and BMP4 signals^68,69^, myofibroblasts and smooth muscle cells around the crypt bottom secrete BMP-inhibitor proteins such as Gremlin 1 and Gremlin 2 that sequester BMPs before they can bind the BMP receptors^70,71^. As epithelial precursors continue to proliferate and move up the crypt, they give rise to the highly proliferative transit amplifying (TA) cell compartment. Finally, differentiation into the destined cell type of for instance the absorptive or secretory lineage occurs under the influence of both environmental and intrinsically programmed factor dynamics^63^.

When aGVHD of the gut develops, the histopathology characteristically demonstrates epithelial apoptosis within intestinal crypts^72,73^. In addition to crypt loss, the number of CBC ISCs per crypt is reduced in experimental GVHD^74–77^. Severe colonic crypt loss at the time of GVHD has been associated with delayed recovery, persistence of symptoms, and the development of SR-GVHD^78^, suggesting an impaired capacity to recover beyond the initial insult. In addition, PCs are reduced in GVHD^76,77,79–83^, which may hamper their ability to provide the indispensable niche factors Wnt3, EGF and membrane-bound Notch ligands for the maintenance of ISC integrity. Besides their role as regulators of ISC proliferation, PCs play an important physiologic role in the production of antimicrobial (AMP) and the release of immunomodulatory proteins (e.g., IgA, IL-1β), both key components of host defense in the gut^84^. Α-defensins are a major class of AMPs produced by PCs, and their production is markedly reduced in experimental GVHD^80,81^, as well as in GVHD patients^83^. Loss of PC α-defensins has been associated with decreased bacterial diversity and domination of bacterial species such as Proteobacteria at the phylum level, Enterobacteriales at the order level, and Escherichia and Bacteroides at the genus level, some of which pathogenic^80,81^. Interestingly, increased plasma levels of the AMP REG3α act as a biomarker of GI-GVHD, predictive of response to therapy, non-relapse mortality and survival^85^. Murine transplant recipients deficient in Reg3γ, the claimed mouse ortholog of REG3α, developed more severe GI-GVHD with increased crypt apoptosis^86^. As such, PC deficiency may contribute to GVHD pathology due to the impairment of ISC niche functions as well as through a reduction in bacterial containment.

Up until recently it was uncertain whether crypt loss in GVHD was directly caused by the effector mechanisms of allo-T cells. Using 3D microscopy, donor T cells were shown to primarily invade the intestinal crypt region early after allo-BMT^77,87^. The potential of allo-T cells to damage the intestinal crypt compartment was studied using so-called intestinal organoid cultures; self-organizing, 3D mini-guts, that form from isolated crypts or purified ISCs when cultured in the presence of defined ISC niche growth factors EGF, Rspo-1 and BMP-inhibitor Noggin^58^. Intestinal organoids contain multiple epithelial cell types, including Lgr5^+^ ISCs, and ex vivo culture models with allogeneic T cells recapitulated in vivo ISC damage^77^. Interferon (IFN)-γ secreted by allo-T cells directly caused ISC apoptosis through signaling via the IFNγ receptor (IFNγR) expressed by ISCs^77^. Interestingly, blocking IFNγR signaling with the JAK1/2-specific inhibitor Ruxolinitib early after allo-BMT protected ISCs from IFNγ-induced damage^77^. Ruxolitinib has recently been approved by the FDA for treatment of SR-GVHD^7^. The rationale for its use in GVHD is based on the suppression of allo-T cell activation, proliferation, cytokine production, and promoting a more favorable regulatory T cell to conventional T cell ratio. The findings of this study indicate that earlier posttransplant use might have a beneficial, target-tissue-protective effect in patients developing GI-GVHD^88^.

Finally, other protective mechanisms downstream of T cell-induced cytotoxicity have recently been described, that could protect epithelial cells in preclinical models. For example, inhibition of HSP90 by 17AAG after allo-HSCT protected the ISC niche^38^. HSP90 is released during tissue damage and induces the intracellular response to ER stress, which PCs are particularly sensitive to^89^. Administration of 17AAG was found to decrease the ER stress actor expression and increase the level of spliced XBP1 important for the regulation of the unfolded-protein response, and preserved both PCs and ISCs in two MHC-mismatched models^38^. A second example concerns serine protease inhibitor 6 (Spi6), the only known endogenous inhibitor of the cytolytic serine protease Granzyme B (GzmB) which protects immune cells from GzmB-mediated damage. In a GVHD model, host Spi6 expression in the non-hematopoietic compartment played a prominent role in GVHD protection, independently of donor-derived GzmB, and Spi6 was upregulated in the intestinal epithelium upon irradiation and subsequent GVHD induction^90^. A third example is found in the inhibitors of apoptosis proteins (IAPs), which are classically involved in the inhibition of cell death proteases such as caspase 3. IAP inhibition was found to exacerbate GVHD, but not when IAP1/XIAP deficiency was limited to the immune system. This suggests intact tissue IAPs are relevant to tissue protection in GVHD^91^. Patients with XIAP deficiency undergoing allo-HSCT after myeloablative conditioning appear to have a poor overall outcome and extra protection against GVHD may be crucial for successful transplantation of these recipients^92^. A fourth example suggests manipulating epithelial integrity regulators to protect the epithelium in GVHD^93^. The expression of MLCK210, an established factor in epithelial tight junction regulation, was found to be increased in the intestinal epithelium of GVHD patients and mice^93^. MLCK210 deficiency in the host led to decreased barrier dysfunction, lower clinical GVHD sores and increased survival in multiple mouse GVHD models. Target-tissue allo-T cell numbers were lower in MLCK210-deficient hosts, and the GzmB-expressing CD8 T cell fraction in mesenteric lymph nodes (MLNs) was reduced^93^. Multiple mechanisms can attribute to this however, including activation, proliferation and homing of the allo-T cells, as well as epithelial–T cell interaction specific factors. Lastly, a recent study describes the disruption of oxidative phosphorylation in IECs exposed to allo-T cells, caused by a reduction in succinate dehydrogenase A (SDHA), a component of mitochondrial complex II^94^. In colonic biopsies from confirmed GI-GVHD patients the amount of SDHA was reduced in comparison to patients suspected of GI-GVHD that could not be histopathological confirmed. Genetically increasing SDHA levels in an experimental GVHD model reduced clinical and histopathological intestinal GVHD severity. Further study of all four approaches is required to establish usefulness in the clinical setting.

#### Preventing T cell trafficking to the gut

Given the epithelial damage caused by allo-T cells, blocking their entry into the intestines provides a promising strategy for tissue protection without increasing global immunosuppression and associated risks of relapse or infection elsewhere. Expression of α4β7 integrin is an important contributor to T cell homing to the GI tract, and plays a major role in the homing of allo-T cells to the GI tract as well^95,96^. Blocking α4β7 binding to its constitutively expressed receptor MAdCAM-1 on intestinal endothelium with anti-MAdCAM-1 antibody after GVHD induction^96^, or using α4β7-deficient donor T cells^95^, selectively reduced CD8 T cell infiltration in the gut^96^ and led to less GI-GVHD^95^. Interestingly, it was recently discovered that MAdCAM-1 expression in the small intestine vasculature localizes predominantly to vessels located in the lower crypt region, offering a possible explanation for the observed pattern of allo-T cells invading the crypt region early posttransplant. As such, inhibition of the α4β7-integrin/MAdCAM-1 axis reduced T cell infiltrate into the crypt base region of the mucosa and protected the ISC compartment from GVHD^87^. In a clinical setting, patients with GI-GVHD had a significantly higher percentage of α4β7-expressing memory T cell subsets than patients with skin-only GVHD or patients with no evidence of GVHD^97^. Retrospective studies indicated potential efficacy of vedolizumab, an anti-α4β7 antibody, for reduction of GI-GVHD severity^98,99^. Results of a prospective, dose-finding trial of vedolizumab starting 1 day prior to transplant are promising; the treatment was well tolerated, and the incidence of subsequent GI-GVHD development was low^100^. Blocking the α4β7 integrin pathway with monoclonal antibodies is therefore a promising strategy for protecting the crypt compartment following allo-HSCT. Antibodies that block only the β7 subunit may hold promise as well. These, in addition to antagonizing the α4β7-MAdCAM-1 mediated T cell influx, also target the αEβ7-E-cadherin interaction, believed to be important for T cell retention in the intraepithelial compartment^101^. A phase II clinical trial of the recombinant human anti-β7 etrolizumab for inflammatory bowel disease had promising results^102^. Future study will have to show if it can be useful in the treatment of GVHD as well.

In addition to protecting the intestinal epithelium from the effector phase of aGVHD, prophylactic inhibition of T cell entry to the gut may also protect against epithelium-dependent contributions to GVHD development. It was recently reported that intestinal epithelial antigen presentation can propagate alloreactive T cell responses. This is contrary the notion that host and donor professional antigen presenting cells (APCs) are the principle APC populations contributing to the activation of alloreactive donor T cells^103^. Despite the dominant role of hematopoietic APCs in propagating MHC-I-restricted/CD8 T cell-dependent GVHD^104^, radio-resistant non-hematopoietic APCs could contribute to the initiation of MHC-I-dependent GVHD as well^105^. Furthermore, profound deletion of professional host APCs did not decrease CD4-dependent GVHD in both a MHC-matched miHA-mismatched and a MHC-II-mismatched mouse model^106^. The action of recipient non-hematopoietic, non-professional APCs was sufficient to induce lethal GVHD^107^. MHC-II expression on IECs specifically could thus initiate lethal GVH immune responses, even in the presence of other types of APCs^108^. As such, approaches to reduce intestinal epithelial MHC-II expression, for instance through the initiation of a high fat diet in mice^109^, may reduce the development of experimental GVHD. Nonetheless, preventing donor T cells from reaching the intestinal epithelium with agents such as vedolizumab may be the most promising approach for reducing intestinal epithelial antigen presentation at the present time.

#### Preventing acute GVHD to prevent chronic GVHD

In some cases acute GVHD can progress or contribute to the development of chronic GVHD (cGVHD)^110,111^, and aGVHD is a well-defined risk factor for cGVHD^12^. While certain aspects of acute GVHD pathophysiology may be shared with cGVHD, such as the involvement of Th17/Tc17^112,113^, there is a paucity in research data studying the links between intestinal epithelial injury and the development of consequent cGVHD of the gut. Most recent insight in the pathobiology include an allogeneic ‘auto-immune’-like course of events, with defective thymic deletion of self-reactive T cells and aberrant B cell activation and production of antibodies^114^. Therefore, new targets of therapy include T and B cell-signaling pathways that are operational during cGVHD^115^. As aberrant tissue repair mechanisms, an inflammatory local milieu and continuous antigen exposure are also contributors in the development of cGVHD^116^, approaches discussed above to prevent intestinal epithelial injury and the development of acute GVHD are in essence applicable in the prevention of cGVHD as well.

### Stimulating epithelial cell restoration

**Fig. 4:**
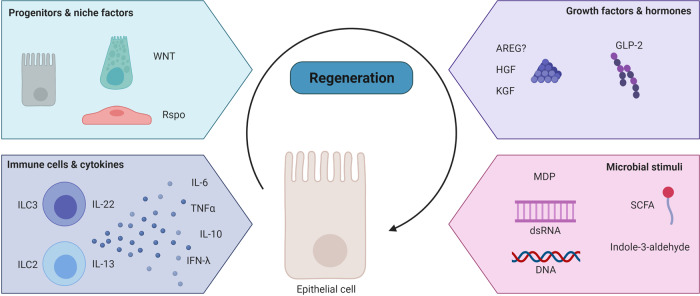
Regenerative treatment options in GI-GVHD. Restoration of the epithelial barrier during the course of GVHD may occur at several levels. The epithelium reconstitutes from within, deriving from progenitors under the influence of supportive niche factors. It can also be supported in its regeneration from its immediate surroundings, for instance through the action of immune cells or particular excreted cytokines, growth factors and hormones. Finally microbial components can contribute to intestinal epithelial healing. Created with BioRender.com.

GVHD treatments have traditionally emphasized immunosuppression, and advances have focused on novel ways to accomplish this. In order to make continued meaningful progress, it is necessary to approach GVHD from additional perspectives. A promising and complimentary approach may be to focus on stimulating epithelial repair. Several processes are involved in the maintenance and recovery of the epithelium, including proliferation and differentiation of intestinal cells, as well as cell migration. Major pathways involved in these processes are multifactorial, and some examples are listed in Table 2. While it has been postulated that GVHD is mainly a disease of the inability to regenerate^8^, evidence exists that regeneration does take place, but may not be enough to overcome the continuous insult. This is for instance illustrated by the fact that intestinal epithelial crypts that survive allo-T cell insult in fact proliferate more than crypts of matched controls^77^. In addition, enterocytes of patients suffering from refractory GI-GVHD showed significant telomere shortening, which is associated with compensatory proliferation^117^. Below we will discuss regenerative approaches that hold promise to support the epithelium in the context of GVHD, either by stimulating epithelial constituents to promote recovery of the lining itself or by influencing the mucosal microenvironment (Fig. 4).

**Table 2:** Major pathways involved in intestinal epithelial regeneration and repair.

Signaling pathway	(S)timulation/(I)nhibition	Effect	Example of eliciting factor and/or mechanism	Ref.
mTORC1/SIRT1	S	ISC expansion	Caloric restriction	^220^
PI3K/AKT	S	IEC proliferation, G1 cell cycle progression	Binding of EGF, TGF-α	^221^
WNT/R-spondin/β-catenin	S	ISC proliferation, suppressed IEC differentiation	Arachidonic acid presence	^67,222^
STAT5/NFκβ	STAT5 S	ISC proliferation, crypt regeneration	Cytokine receptor activation	^223^
	NFκβ I	Mucosal wound healing	Decreased MLCK phosphorylation and TJ permeability	^224^
Hippo/YAP-TAZ	Hippo I, YAP S	Intestinal regeneration in DSS colitis	Binding of stroma-derived Immunoglobulin superfamily containing leucin-rich repeat protein (ISLR)	^225,226^
		Low Wnt signaling, wound-healing response		
		Excessive PC differentiation, crypt regeneration		
		Increased organoid growth	Binding of bile acids to TGR5	^227^
	Hippo S, YAP I	Maintenance Wnt signaling, canonical stem cell function	–	^228^
SMAD	S	Increased barrier function through TJ protein upregulation	Binding of TGF-β	^229^
BMP/SMAD	I	ISC maintenance, expansion	Relief of direct, HDAC1-mediated transcriptional repression of stem cell signature genes	^230^
ERK/MAPK	S	ISC expansion, crypt formation, IEC proliferation	Binding of HGF to MET	^175^
		Increased barrier function through TJ protein upregulation	Binding of TGF-β	^229^
		Enhanced IEC migration	Binding of Flagellin	^231^
STAT3	S	Intestinal mucosa regeneration, organoid formation	Downstream FAK activation and integrin signaling	^148^
		ISC expansion, crypt formation, organoid proliferation	Binding of IL-22 to IL-22R	^76^
Myd88/NFκβ	S/I	Regulation of intestinal epithelial integrity and inflammatory responses	NFκβ inhibition leads to severe chronic inflammation and epithelial apoptosis	^232^
			Epithelial MyD88 required for survival in multiple colitis models	^233^
c-Jun/AP-1	S	Promotion of epithelial restitution after wounding through cell migration	Upregulation of PLCγ1-induced Ca^2+^ signaling	^234^
JNK2	S	Epithelial barrier maintenance, enhanced Goblet cell and EEC differentiation and mucus production	Protection from DSS colitis, reduced barrier dysfunction and enterocyte apoptosis, increased Atoh1 expression	^235^

#### Restoration from within the epithelial compartment

Despite growing insights into ISC maintenance under homeostatic conditions, the principles underlying epithelial regeneration for maintenance of barrier function after tissue damage remain incompletely understood. Although radiation injury can cause a significant loss of ISCs, the Lgr5^+^ CBC cell pool is relatively resistant to radiation injury, reportedly due to their ability to repair DNA damage^118^. Crypt repopulation originated from surviving CBC cells^118^, which are essential, as Lgr5 genetic deletion and subsequent irradiation severely hinders the regenerative response^119^. There appears to be considerable plasticity in intestinal progenitor cells in response to damage. Upon CBC ablation, progenitors were able to dedifferentiate and regain stemness, thereby replenishing the ISC pool and subsequently the mature enterocytes at the epithelial surface^120^. Both secretory^121,122^ and enterocyte^123^ progenitors are capable of this reversion, and even fully differentiated enterocytes can contribute to crypt repopulation under specific circumstances of extreme damage^124^. In both instances, the expression of Lgr5 reappeared at the base of the crypt^121,123^, preceded by the re-expression of the ISC-restricted transcription factor Ascl2^125^. Even a subset of Paneth cells acquired multipotency upon irradiation through Notch activation^126^. The reprogramming of adult differentiated cells appears to have a developmental link^122^, as fetal mouse IECs can give rise to the adult ISC pool irrespective of their location or Lgr5 status^127^. Additionally, during infectious insult fetal-type gene expression programs play a role in epithelial recovery, as the murine ISC niche can revert to a fetal-like state upon parasitic helminth infection^128^. The importance of these complex crypt stem cell and progenitor dynamics in regeneration during GVHD-induced damage is currently unknown. The prolonged damage to the GI tract present in GVHD likely includes substantial insult to the cells with regenerative potential that are responsible for overall epithelial reconstitution.

#### Restoration through replenishment of ISC niche factors

Several niche factors secreted by cells in the microenvironment of the crypt compartment could contribute to restoration of crypt damage in allo-HSCT. Wnt signaling is essential for ISC maintenance, with cytoplasmic β-catenin translocating to the nucleus, interacting with transcription factors of the TCF/LEF family, and subsequently activating expression of target proteins involved in proliferation, such as Myc^67^. Wnt is required for crypt regeneration after damage^129,130^ and during inflammation, as seen in a DSS colitis model^131^. Short term Wnt agonism has been proposed as a therapeutic countermeasure against irradiation-induced gastrointestinal damage in mice^132^. GSK3β is an essential kinase of the Wnt/β-catenin pathway involved in the control of the cytoplasmic levels of β-catenin and its inhibition increases β-catenin availability and downstream Myc expression^133^. In an observational pilot study the known GSK3β-inhibitor lithium was used to salvage SR-GVHD, with promising results^134^.

Another approach to potentiate the Wnt pathway in the experimental transplant setting is through R-spondin-dependent modulation of Lgr5 signaling. It has recently been proposed that the most abundant R-spondin in the intestines, Rspo-3, is predominantly produced by lymphatic endothelial cells (LECS) in the lamina propria. LECS were found to be reduced in number and their Rspo-3 production impaired in experimental GVHD^135^. Exogenous administration of Rspo-3 promoted stem cell recovery and epithelial regeneration in the colon in a murine DSS-induced colitis model^124^. Another source of R-spondins are the recently described MAP3K2-regulated intestinal stromal cells at the bottom of colon crypts, which release Rspo-1 to maintain Lgr5+ ISCs during DSS colitis^66^. A prophylactic strategy of enhancing Wnt signaling with administration of Rspo-1 reduced murine colon pathology resulting from radiation^136^ and chemotherapy injury^136,137^. Furthermore, in a MHC-mismatched allogeneic BMT model, pretransplant treatment with Rspo-1 was associated with increased Olfm4^+^ ISCs and reduced GVHD mortality^75^. In addition, Rspo1 administration stimulated differentiation of ISCs towards PCs, increasing their numbers, with a positive impact on the secretion of luminal α-defensins and microbiome diversity^81^. Rspo administration might therefore be a promising approach, but future clinical studies are necessary to investigate efficacy and safety.

Also other stem cell niche factors are important for crypt regeneration and intestinal epithelial integrity after damaging or inflammatory insults. For instance, mice with impaired EGF-receptor signaling in the gut were more susceptible to inflammation in DSS colitis, due to impaired IEC regeneration and consequent barrier compromise^138^. In a phase I study, patients receiving urinary-derived human chorionic gonadotropin (uhCG) that could supplement EGF as supportive care in aGVHD had a promising biochemical response correlating with day 28 clinical response^139^. Also Notch ligands like Jag1 and DLL3 have been implicated in intestinal epithelial reconstitution and proliferation during inflammation, downstream of YAP pathway activation^140^, but have thus far not been studied in the context of GVHD.

#### Restoration through the regulation of immune cells & cytokines

In addition to epithelial and stromal contributions to the ISC niche, there is a growing appreciation that the local immune system can regulate the ISC compartment and its regeneration^141^. The IL-10-type cytokine IL-22^142^ is produced by a variety of immune cells and is involved in antimicrobial immunity and in both induction and resolution of inflammation in the intestine^143–145^. In addition, IL-22 has been implicated in the maintenance of the intestinal barrier and epithelial repair, due to its influence on mucus production^145^ and IEC proliferation^76,146^. IL-22 derived from group 3 ILCs (ILC3s) was shown to be protective in the GI tracts of transplant recipients in experimental GVHD^74^. However, the pathophysiological process of gut GVHD leads to loss of intestinal ILC3s and their protective IL-22 production^74^. Furthermore, patients with low numbers of ILCs in circulation prior to transplant had an increased risk of developing GVHD^147^. Interestingly, in vivo treatment of transplanted mice with the recombinant human IL-22 dimer/Fc fusion molecule F-652 (Generon Corp., Shanghai) reduced GVHD-related clinical scoring and mortality in a MHC-matched GVHD model^76^. IL-22 activated STAT3 phosphorylation in small intestine ISCs and organoids, promoting ISC survival and expansion as well as overall epithelial regeneration and recovery^76^. Findings of the role of the IL-22-STAT3 axis in crypt regeneration have been validated by the fact that STAT3 was required for damage-induced crypt regeneration after radiation injury^148^. In addition, IL-22 was required for an effective DNA damage response in protecting ISCs from genotoxic stress^149^. The influence of IL-22 on crypt regeneration under homeostatic conditions in vivo has not yet been studied. A Phase II trial for treatment of newly diagnosed GI-GVHD with a combination of corticosteroids and a recombinant human IL-22 dimer has recently been performed to investigate the safety potential of this novel tissue-regenerative approach to GVHD treatment (NCT02406651).

While therapy with IL-22 appears promising, additional immune-mediated pathways of regeneration may hold translational potential as well. IL-22-independent effects of ILC3s on epithelial regeneration involving the Hippo-YAP1 pathway have been described after methotrexate-induced GI damage^150^. The authors proposed a dichotomy between stem cell maintenance, which could be ILC3/IL-22/STAT3 dependent, and crypt proliferation, which they found to occur in a ILC3-dependent but IL-22 independent manner^150^. As such, the application of an ILC3-based cell therapy, instead of only administering IL-22, may have additional benefits for GVHD patients.

Also another type of innate lymphocyte cells, ILC2s residing in MLNs and Peyer’s patches, are known to support the intestinal barrier function, by inducing Goblet cell expansion through IL-13 secretion in response to Tuft-cell-derived IL-25^151^. Goblet cells are important for barrier function by secreting mucus that shields the intestinal epithelium from gut contents and microbes, and were found to be reduced in GVHD in mice and patients^13^. As mentioned earlier, ILCs are lost in GVHD, but pretransplant administration of IL-25 led to protective Goblet cell induction, decreased bacterial translocation, and ameliorated GVHD, increasing survival in a haploidentical and MHC-mismatched model^13^. In addition, ILC2-derived IL-13 may have a direct regenerative effect through binding the IL-13R expressed on ISCs. IL-13 increased ISC self-renewal and β-catenin signaling^152^.

Recently, it was reported that type III IFNs (IFN-λ), known for their role in epithelial viral defense, have an epithelial protective effect in experimental GVHD^153^. In vivo treatment of naïve mice with recombinant IFN-λ in the form of PEGylated (PEG-)IL-29, which has been tested in phase I-III clinical trials as an adjunctive treatment for hepatitis C virus, increased ISC numbers and led to more efficient ISC-derived organoid growth ex vivo. In experimental GVHD, prophylactic PEG-IL-29 administration prolonged survival, reduced GVHD severity and increased epithelial proliferation^153^.

Some other, classic pro-inflammatory cytokines have been shown to also play a role in maintaining epithelial integrity. Many of these cytokines may be inhibited by immunosuppressive GVHD therapies. For instance, TNFα has long been implicated in GI-GVHD pathogenesis^154^, but also has epithelial-supportive effects in vitro. Low-dose TNFα increased the number of human fetal intestinal organoids, while higher doses impaired organoid formation^155^. Mechanistically, TNF treatment directly promoted Wnt/B-catenin signaling^156^ and increased the expression of several stem cell markers in murine intestinal^156^ and human fetal intestinal organoids^155^, including Acl2. This epithelial-supportive mechanism provides insights as to why TNFα-blockade has had inconsistent results in the treatment of GI-GVHD^157^. A similar paradox can be found with the pro-inflammatory cytokine IL-6. IL-6 inhibition through blockade of IL6R-signaling with tocilizumab has had some promising results in both experimental and clinical GVHD, and has been associated with induction of allo-T cell suppressive Tregs^158,159^. However, IL-6 administration in healthy mice has been associated with STAT3-induced epithelial regenerative effects such as increased intestinal villus height, elongated enterocyte lifespan and a concurrent decrease in pro-apoptotic caspase activity^160^. Accordingly, in a GI damage model of mechanical wound injury, IL-6 inhibition resulted in impaired healing due to decreased proliferation^161^. As such, care should be taken with IL-6-blocking therapeutic approaches in GI-GVHD.

An additional example of cytokine-mediated restoration can be found in the regulatory cytokine IL-10. IL-10- and IL10R deficiency are known to cause severe intestinal disease in both mice and humans^162^, and disruption of the IL-10 signaling pathway resulted in exacerbation of experimental GVHD^163^. Nonetheless, treatment of IL-10 or co-culture with peripherally induced Tregs led to the expansion of ISC numbers in murine organoids, and increased clonogenicity after passage^164^. In addition, human recombinant IL-10 was shown to promote intestinal epithelial proliferation by activation of CREB signaling^165^. Despite the fact that treatment of GVHD by exogenous IL-10 does not seem to be clinically feasible due to its pleiotropic and divergent effects^163^, there may be a crypt-protective effect if epithelial-targeted administration would be possible.

#### Restoration through use of growth factors and hormones

In addition to previously discussed niche factors, stromal cells surrounding the epithelial crypts are important sources of EGF-like growth factors for the intestinal epithelium. Keratinocyte growth factor (KGF) is one of the most well-studied for its protective role during conditioning-induced damage and oral mucositis^166^. While initially described as a growth factor for skin epithelium, KGF can enhance intestinal epithelial proliferation^167^, and crypt cell survival after irradiation^168^. In experimental GVHD, KGF administration started prior to and continued after the transplant reduced GVHD mortality and severity in the GI tract^169,170^. However, administration of palifermin, a recombinant human KGF, did not reduce GVHD incidence or improve overall survival in allo-transplant patients in two randomized controlled trials^171,172^, although it did reduce mucositis incidence and severity in a subgroup of patients^171^. Another EGF-like growth factor in preclinical development is the potent liver mitogen Hepatocyte Growth Factor (HGF) produced by intestinal fibroblasts and macrophages^173^. Using a human HGF expression vector injected into muscle at the time of transplant, stable expression of hHGF in HSCT recipient mice reduced GVHD histopathology and crypt apoptosis^174^. Interestingly, HGF was found to be a possible substitute for EGF in intestinal organoid cultures. Mice lacking the receptor for HGF in their epithelium had reduced numbers of proliferating crypts and ISCs after irradiation^175^. Perhaps the protective effect of HGF in experimental GVHD is a result of directly targeting the intestinal epithelium. Lastly, it has been postulated that amphiregulin (AREG), a weak EGF-receptor agonist produced by a multitude of immune, stromal and epithelial cells, may have intestinal epithelial regenerative effects in the GVHD setting. It has been implicated as a possible plasma biomarker for risk stratification and steroid response in aGVHD^176^. Genetic disruption of Areg significantly impaired intestinal regeneration after radiation injury in full knockout mice^177^. Nonetheless, the beneficial effects of AREG on experimental GVHD incidence and mortality observed thus far do not directly implicate epithelial regeneration as the main mechanism and could still be ascribed to allo-immune suppression, such as through Treg function enhancement^178–180^. Taken together, the intestinal regenerative effect of growth factor substituents may be promising, but seems to have limited application in the clinic thus far.

In addition to growth factors, enteroendocrine hormones may have intestinal epithelial protective effects^181^ in the context of GI-GVHD. Glucagon-like peptide (GLP)-2 is produced by intestinal L-cells, which are a subset of enteroendocrine cells. L-cells are reduced in mice and patients that develop GVHD^182^. In vivo, GLP-2 agonism acutely increased the proportion of Lgr5+ ISCs in S-phase and prolonged treatment increased numbers of Olfm4+ ISCs per crypt^183^. GLP-2 stimulation of intestinal organoids led to increased organoid size^182^. Prophylactic treatment with a GLP-2 agonist injected subcutaneously in a MHC-mismatched mouse model, improved survival, decreased gut GVHD histopathology scores, and restored ISC loss, even when applied as an additive to steroids^182^. Future clinical studies will have to investigate its utility in clinical GVHD patients.

#### Restoration through the supply of protective microbial stimuli

Despite the pro-inflammatory effects of some innate immune signaling pathways, it was demonstrated that specific innate pattern recognition pathways can exert a protective effect on the intestinal epithelium during GVHD in mice. The RIG-I/MAVS pathway is involved in the sensing of dsRNA during infection, while the cGAS/STING signaling pathway is involved in the recognition of DNA. Perturbation of these innate pathways with genetic STING knockouts, changed the sensitivity to GVHD with contrasting effects on outcomes depending on the donor/recipient disparity and the specifics of the transplant models utilized^184,185^. In a MHC-mismatched model, treatment with 3pRNA or DNA prior to allo-HSCT protected mice from conditioning-induced intestinal damage and GVHD without diminishing the GVL activity of allo-T cells. Mechanistically, activation of the pathways led to the expression of protective type I IFNs (IFN-Is), which were indispensable for the maintenance of gut epithelial barrier integrity, but only when they were induced prior to the TBI insult. Treatment of intestinal organoids with 3pRNA and DNA confirmed the direct epithelial effects with increased IFN-I-dependent proliferation^184^. Intestinal epithelial IFN-I signaling was recently implicated in the regulation of stemness and differentiation into secretory-cell lineages. Mice lacking IEC Interferon regulatory factor 2, which downregulates IFN-signaling, had fewer ISCs, accumulation of immature PCs and impaired regeneration after damage^186^. In clinical studies, treatment with IFN-α before HSCT^187^ or after relapse post-HSCT^188^ was associated with a higher incidence of overall acute GVHD. Tight regulation of IFN-signaling induction during injury thus appears to be crucial.

Another cytosolic innate immune pathway implicated in the protection against intestinal epithelial injury is NOD2. It binds to the peptidoglycan muramyl dipeptide (MDP), which is produced by most bacteria. In a T cell-induced enteropathy model, NOD2 deficiency outside the intestinal epithelial compartment led to more severe crypt damage, apoptosis and delayed epithelial regeneration^189^. Similarly, in mouse BMT models, host NOD2 expression in the hematopoietic compartment is protective against the development of GVHD^190^. Nevertheless, MDP was shown to directly increase organoid-forming potential of intestinal crypts and to protect ISCs from oxidative-stress-induced cell death^191^. NOD2 also supported intestinal crypt survival and regeneration after irradiation, both in organoid cultures of NOD2 knockout mice and in vivo^192^. Given these findings, a non-hematopoietic protective role of NOD2 signaling in GVHD protection may also be possible. Nonetheless, more study is required to appreciate whether these mechanisms can also be exploited in GVHD patients to promote regeneration.

Over the past decade, several bacterial metabolic products have been associated with gut barrier integrity, including in the context of GVHD. Many studies have focused on short chain fatty acids (SCFAs), such as butyrate and propionate. Butyrate contributes to intestinal health in multiple ways^193–195^. It was found to directly increase epithelial regeneration in 3D organoid cultures^196^ and improve wound healing through tight junction protein upregulation^196,197^. In the setting of GVHD, intragastric administration of butyrate to allogeneic recipients improved IEC junctional integrity, decreased expression of apoptotic proteins in IECs, and led to decreased GVHD, independent of the induction of Tregs^196^. Clostridia commensals are known butyrate producers, and administration of a microbial cocktail including 17 strains of Clostridiales elevated intraluminal butyrate concentrations, decreased GVHD clinical scores, and increased survival in a mouse GVHD model^196^. This could explain why the presence of Clostridiales^198^ and its protection in the microbiome by selective antibiotic use was found to be associated with reduced GVHD-related mortality in clinical studies^199,200^. It also provides a rationale for the use of fecal microbiota transplants for the treatment of GVHD^201–203^. Recently, it was demonstrated that signaling through non-hematopoietic GPR43, a metabolite sensor, is critical for the GVHD treatment effects of SCFAs, independent of baseline microbiota constitution^204^. Given the clear associations of microbial constituents and GVHD outcomes, manipulation of the enteric flora or associated metabolites represent promising approaches for clinical prevention and treatment of GVHD.

The tryptophan catabolite indole and its derivatives are other product of commensal bacteria with gut immunomodulatory effects. *Lactobacillus-*derived indole-3-aldhehyde, for instance, engages the aryl hydrocarbon receptor (AHR), an environmental sensor and crucial transcription factor for ILC3s in the gut. As such, it can expand ILC3s and their IL-22 production in the intestinal mucosa^205^, as well as influence the immune response via many other immune cell types^206^. AHR ligation however also has a direct epithelial effect, as it was shown to regulate ISC differentiation and thereby maintain barrier integrity^207^. In a GVHD mouse model, administration of indole-3-aldehyde reduced GVHD severity, intestinal epithelial damage, and gut bacterial translocation. The effects were mediated through an IFN-I response observed at the transcriptional level in whole gut samples^208^. In allo-HSCT patients, higher levels of urine 3‐indoxyl sulfate, an indole-derived metabolite, correlated with lower treatment-related mortality and higher overall survival^209^. Therefore, indole-3-aldehyde administration represents another potential interventional approach of interest for treatment of GVHD.

## Concluding remarks and future prospects

The intestinal epithelium experiences substantial toxicity during the course of allogeneic transplantation. Given the pivotal role of alloreactive T cells in GVHD pathogenesis, effective immunosuppression is the cornerstone of GVHD treatment strategies. However, in addition to control of the alloreactive immune response, development of target-organ-focused strategies that can protect the epithelium and stimulate its regeneration is important for further progress in improving clinical outcomes for transplant patients. Given advancements in both experimental and clinical research, it is possible that this hope may be realized in the near future.

Most epithelial-targeted factors and pathways discussed here have pleiotropic effects with complex feedback mechanisms in multiple tissues and different cell types. The mere stimulation or inhibition on the systemic level therefore may not result in the intended outcome. New ways to specifically target the intestine, for example through intestine-directed genetically engineered cells^210^ or carriers such as nanoparticles^211–213^, might make additional GI-targeted approaches more feasible in the future, similar to the wide use of oral budesonide for more targeted administration of corticosteroids to the GI tract. On an even smaller scale, a better understanding of the structural design of factors involved may enable the decoupling of protective functions from pro-inflammatory effects. In a recent study authors were able to design a STAT3-biased IL-22 receptor agonist, which elicited tissue selective STAT3 activation in vivo^214^.

A combinatory approach of factors that hold promise in preliminary trials at different levels of epithelial support could be considered (Table 3). To have an effect, adequate timing of the different strategies will be essential (Fig. 2). Wider use of drug-exposure-targeted pretransplant conditioning to limit the initial damage^29–31,33^ in combination with early initiation of Jak1/2 inhibition to shield the epithelium from allo-T cell-derived IFNy and protect ISCs^77^ may represent a currently attainable approach to improve GVHD prophylaxis. Should GVHD develop, pro-regenerative therapies such as IL-22 could be administered in the front-line setting along with corticosteroids to promote epithelial recovery^76^. Attention must also be paid to maintaining a supportive enteric microbial environment, including preservation of healthy anaerobic commensals such as butyrate producers^199,200^. A comprehensive approach involving these strategies as well as the implementation of additional tissue-targeted modalities currently in development will be necessary to fully incorporate epithelial biology into GVHD treatment strategies and optimize outcomes for HSCT patients.

**Table 3:** Ongoing trials aimed at protecting or regenerating the intestinal epithelium in GI-GVHD treatment or prevention (per February 1st, 2022).

Trial agent	(Proposed) mechanism of action	Phase	Trial number
Reducing DAMPs or response to DAMPS			
Alpha-1 Antitrypsin (AAT)	Serine protease inhibitor degrading heparan sulfate	III	NCT04167514
		II/III	NCT03805789
Blocking alloreactive T cell influx to the gut			
Vedolizumab	α4β7-integrin inhibitor	III	NCT03657160
Natalizumab	Selective α4 subunit adhesion molecule inhibitor	II	NCT02133924
Blocking cytokine-mediated killing			
Ruxolitinib	JAK1/2 inhibitor	II	NCT04384692
		II	NCT04061876
		II	NCT03701698
		I/II	NCT03491215
		IV	NCT02386800
		I	NCT05121142
Baricitinib	JAK1/2 inhibitor	I	NCT04131738
Pacritinib	JAK2 inhibitor	I/II	NCT02891603
Itacitinib	JAK1 inhibitor	I	NCT04070781
		II	NCT03846479
		I	NCT03755414
Tocilizumab	IL-6 inhibitor	II	NCT04395222
		I	NCT04070781
		II	NCT03434730
		II	NCT04688021
Jaktinib	JAK1/2/3 inhibitor	II	NCT04971551
TQ05105	JAK2 inhibitor	I/II	NCT04941404
Regeneration of the epithelium			
Pregnyl	human Chorionic Gonadotrophin (hCG)/EGF	I/II	NCT02525029
		I/II	NCT05123040
IL-22Fc	IL-22R binding	Ib	NCT04539470
Lactobacillus Plantarum	Producers of indole-3-aldhehyde	III	NCT03057054
Galacto-oligosaccharide	Prebiotic sustaining butyrate-producing bacteria	I/II	NCT04373057
